# Supplementary figures and images for: NMR metabolomics-guided DNA methylation mortality predictors
Source: eBioMedicine. 2024 Aug 17;107:105279. doi: 10.1016/j.ebiom.2024.105279 (PMC11378104; doi:10.1016/j.ebiom.2024.105279)

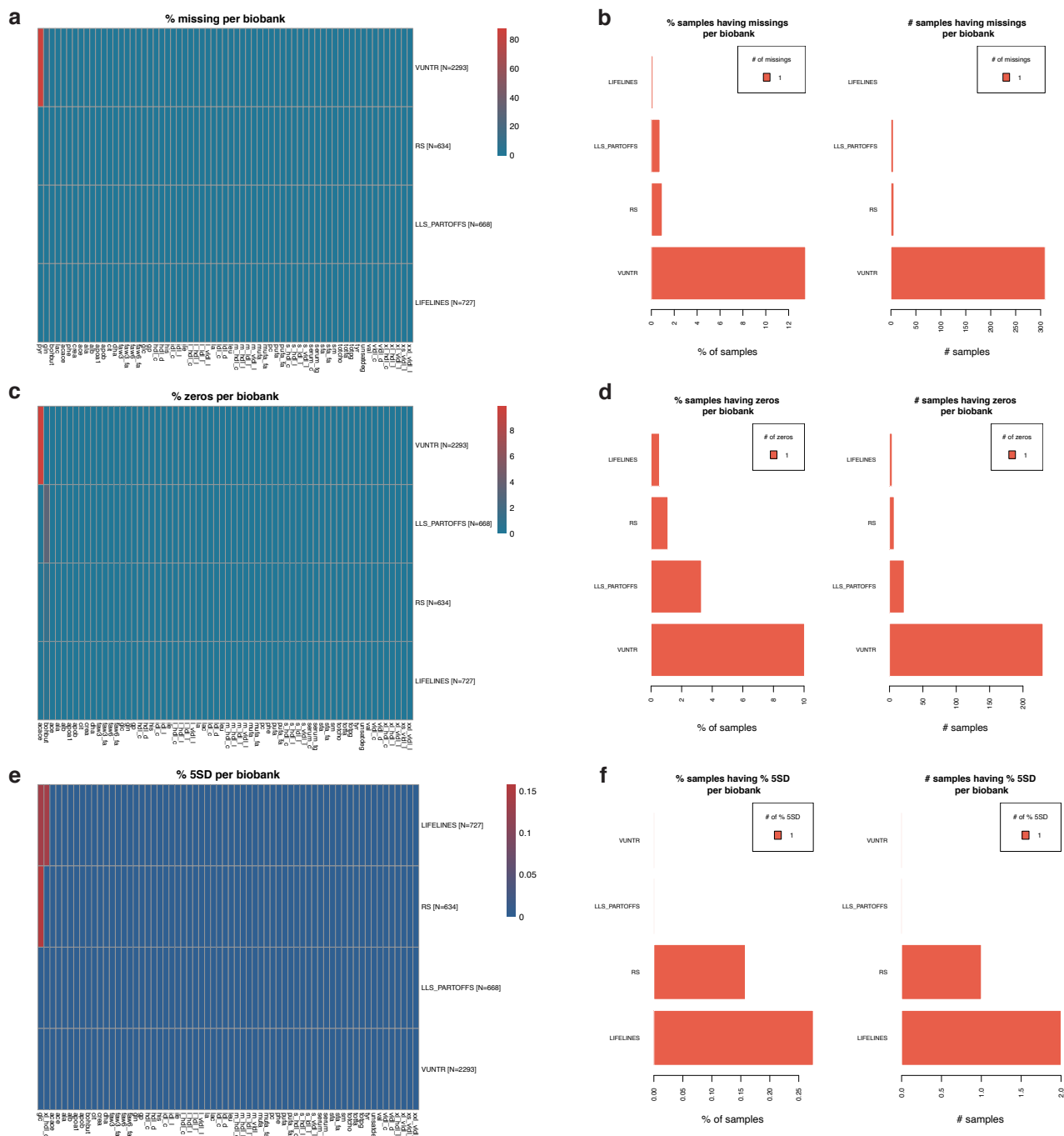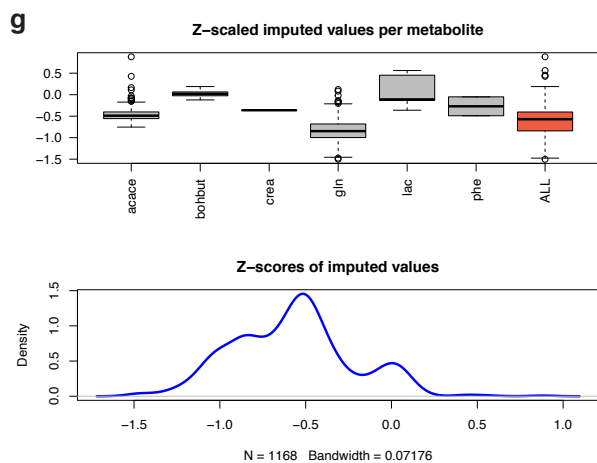

Supplement: Figure S1 — Preprocessing of the metabolomics dataset. Percentages of a) missing values. c) zeros, and e) outliers, in each 64 metabolomics features in the 4 BIOS cohorts (VUNTR, RS, LLS_PAROFFS and LIFELINES). Bar-plots representing the number of b) missing values, d) zeros, and f) outliers in the samples divided per cohort. Finally, g) indicates the Z-score distributions of the 1168 imputed missing values in the dataset. [file mmc1.pdf]

## Uncalibrated metabolites

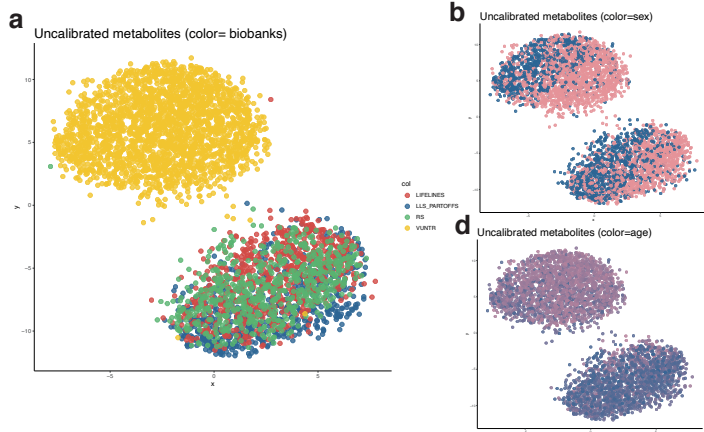

## Calibrated metabolites

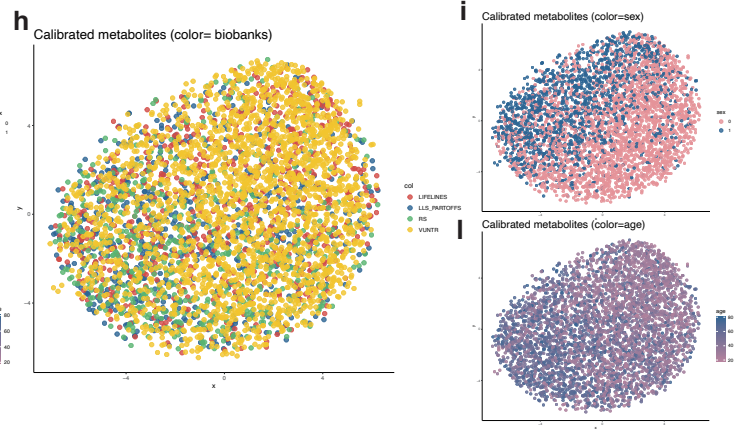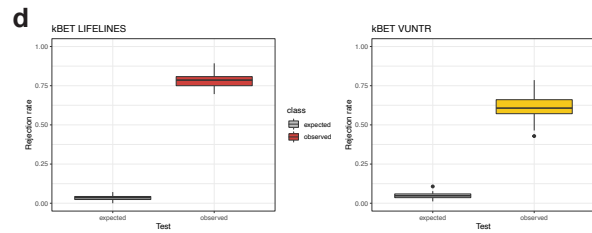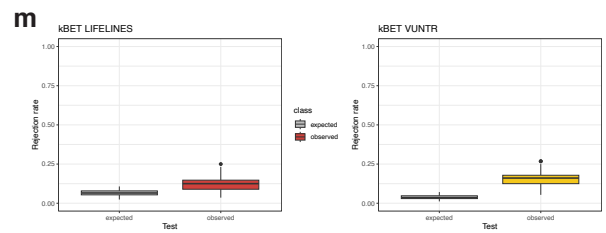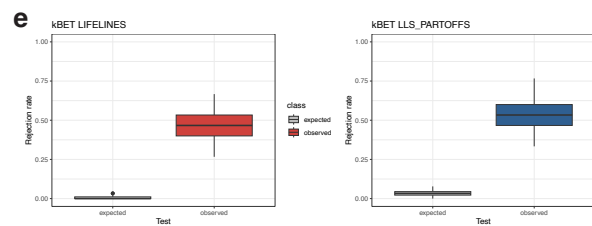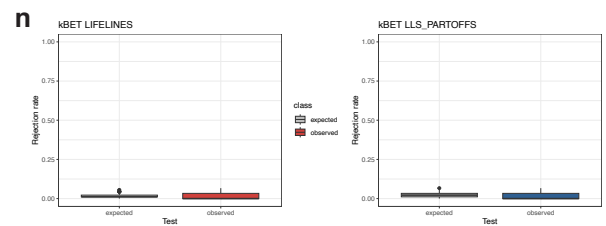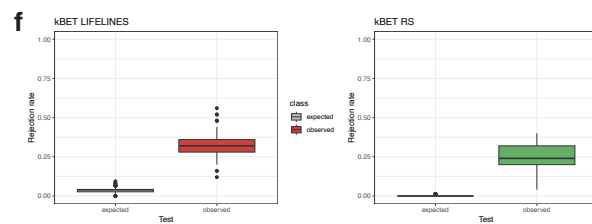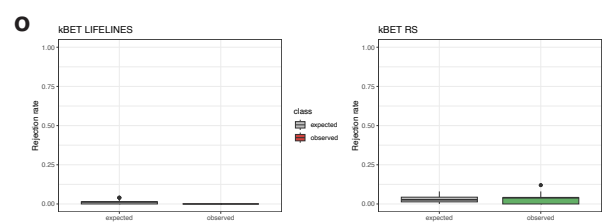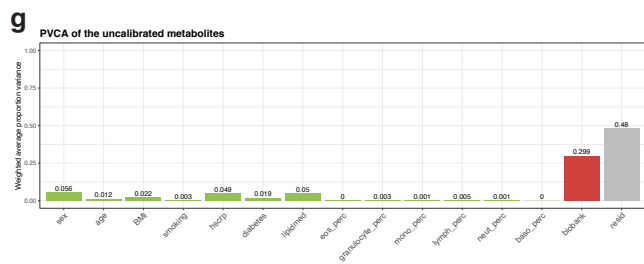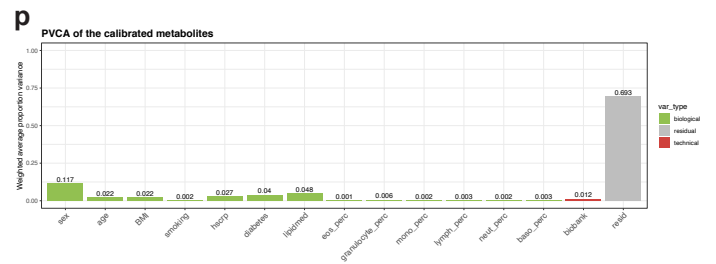

Supplement: Figure S3 — Comparisons of the metabolomics datasets before and after the calibration. tSNE plots coloured by biobanks, sex, and age before (respectively a, b, c) and after (respectively h, i, l) the calibration. kBET shows an improved mixing in the matching samples between LIFELINES and VUNTR (d before and h and after), LLS LIFELINES and LLS_PARTOFFS (e before and n after), and RS and LIFELINES (f before and o after). PVCA before g) and after p) calibration. [file mmc3.pdf]

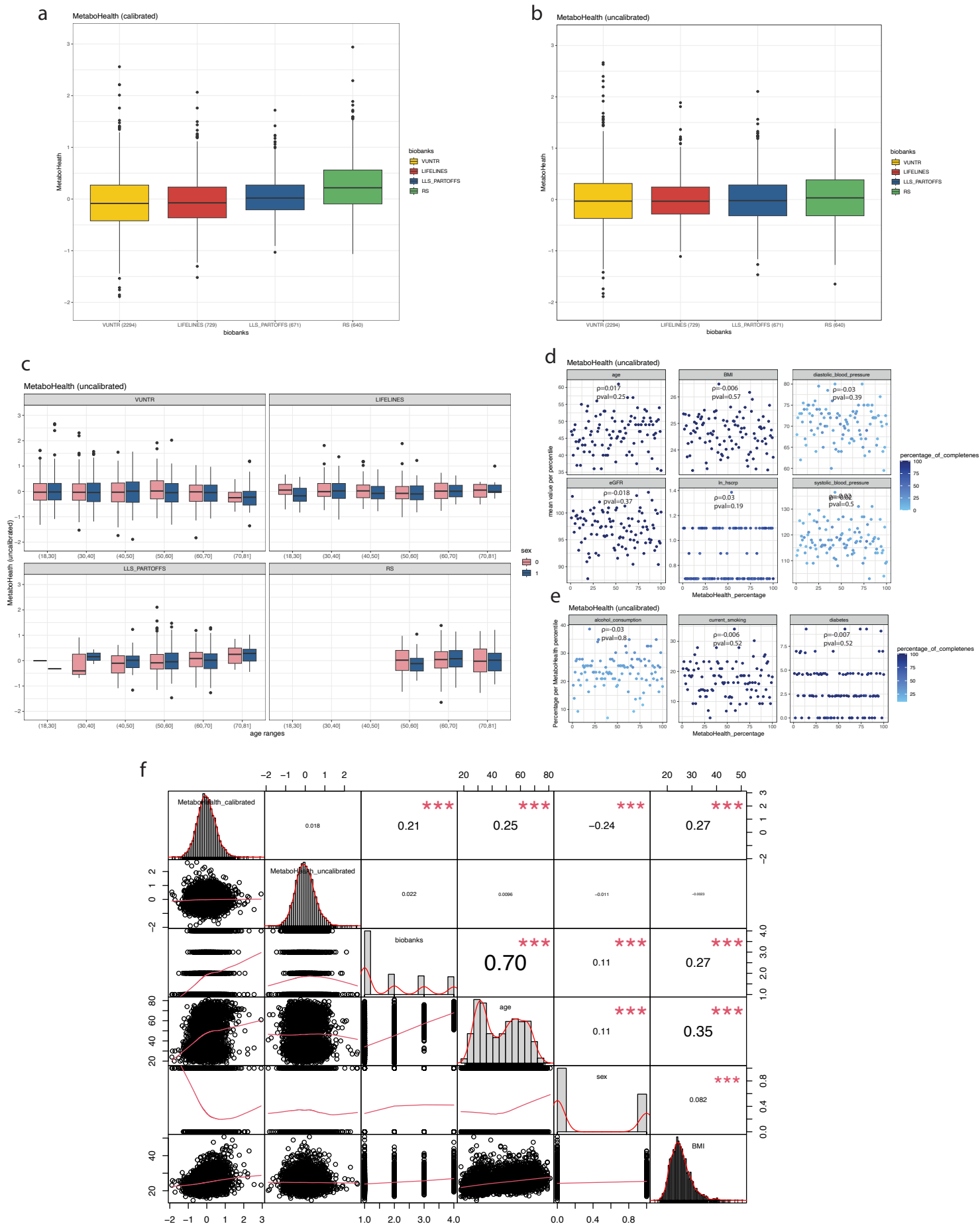

Supplement: Figure S4 — Calibrated and uncalibrated MetaboHealth. Box-plot comparing the a) calibrated and b) uncalibrated MetaboHealth values in each biobank, c) Bar-plots showing the differences in men and women in the uncalibrated MetaboHealth in the 4 cohorts. d) Observed mean values of age, BMI, eGFR, hsCRP and pressure and e) alcohol consumption, current smoking, and diabetes ordered following the uncalibrated MetaboHealth percentile over the entire BIOS population. On top the Spearman correlations (ρ) and its p value f) Correlation chart comparing the calibrated and uncalibrated MetaboHealth, with age, sex, BMI and biobanks. The upper triangle part of the figure indicates the mutual correlations and their relative p value. [file mmc4.pdf]

\_\_\_\_\_

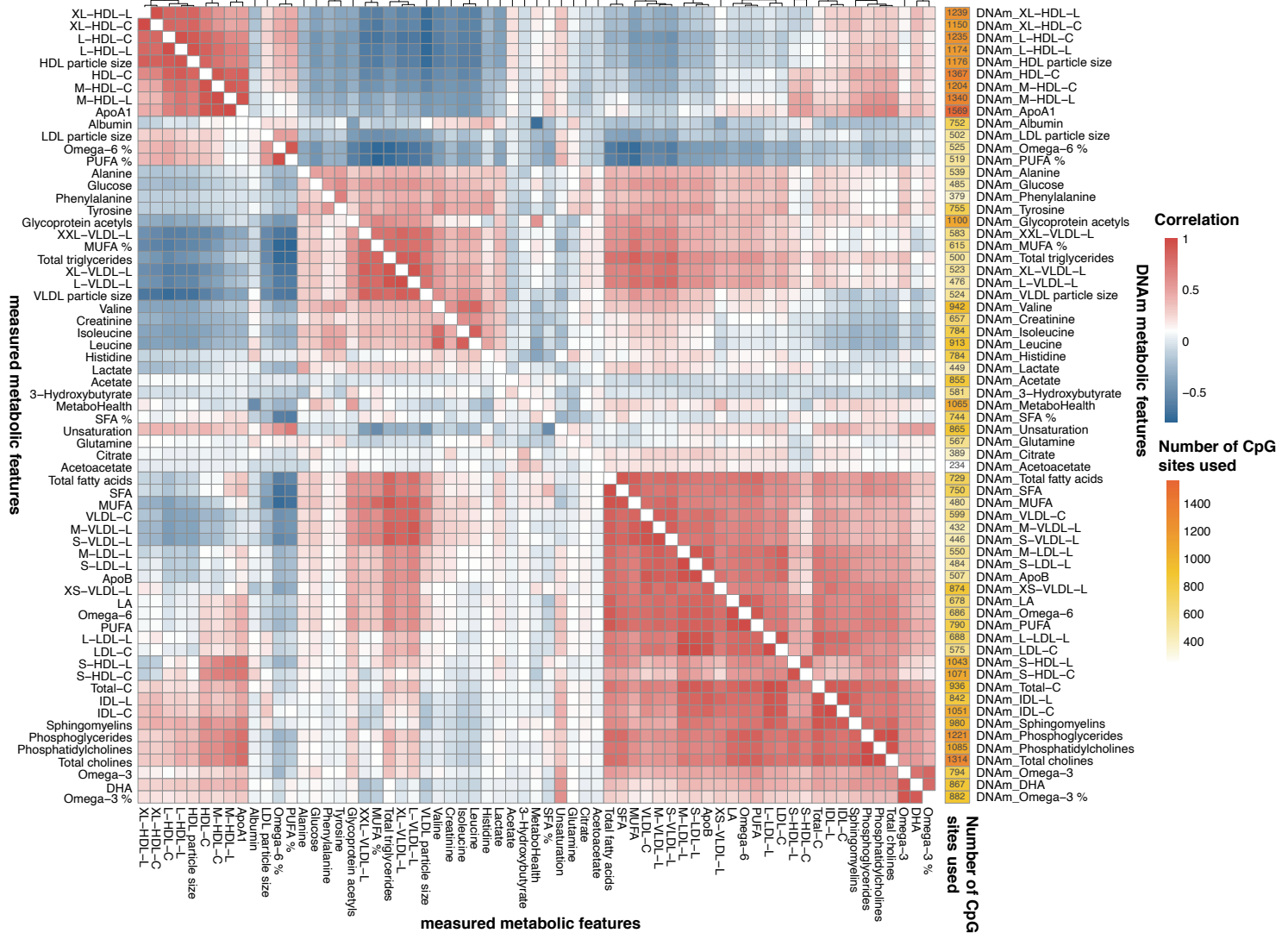

Supplement: Figure S6 — Metabolites intercorrelations and DNAm metabolites intercorrelations. Intercorrelations of the DNAm metabolomics features. A) Clustered intercorrelations between the DNAm metabolic features in the upper triangle and intercorrelations between the measured metabolomics features in the lower triangle. On the right there is also an heatmap indicating the number of CpGs used per model. [file mmc6.pdf]

Supplementary Figure 7: Univariate mortality associations in RS

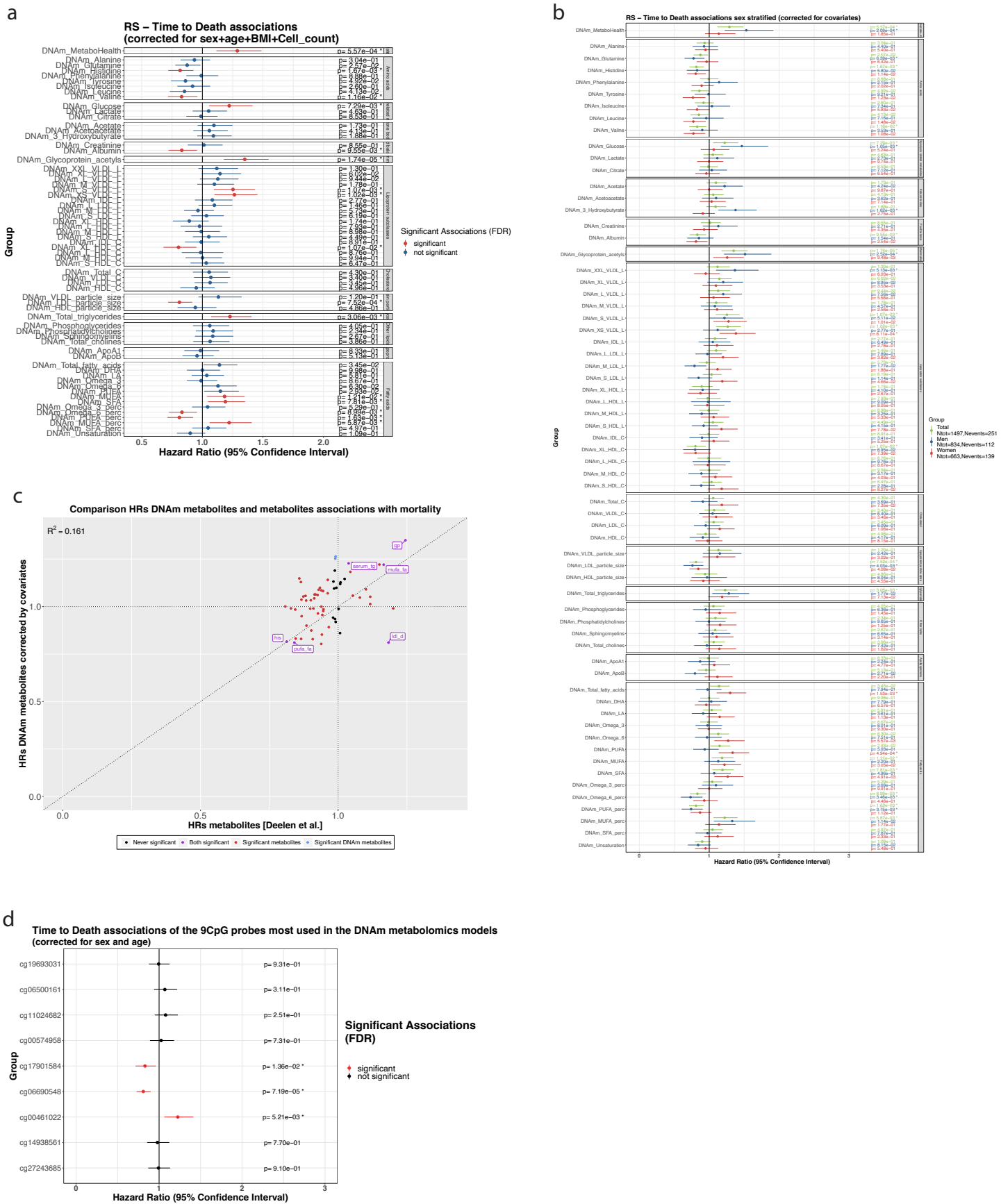

Supplement: Figure S7 — Univariate mortality associations in RS. a) Complete Univariate associations of the DNAm metabolomics features with time to all-cause mortality in RS (N = 1542 with 285 reported deaths). The associations are grouped based on the metabolomics groups and coloured by the significant associations or the metabolites with mortality in Deelen et al. b) Univariate associations of the DNAm metabolomics features split for sex. c) Comparisons of the univariate DNAm metabolomics associations to mortality to the associations of the metabolomics features in Deelen et al. d) Univariate mortality associations of the most used CpG sites in the DNAm metabolomics features. On the right side of each forest plot there are the p values for each univariate association [cox regression]. [file mmc7.pdf]

a

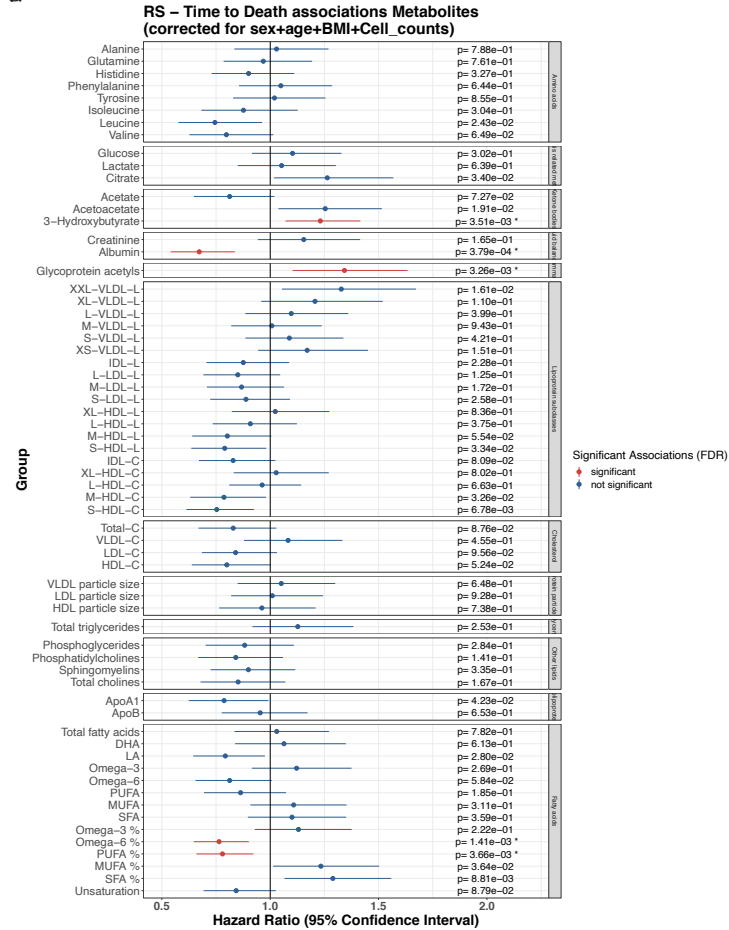

b

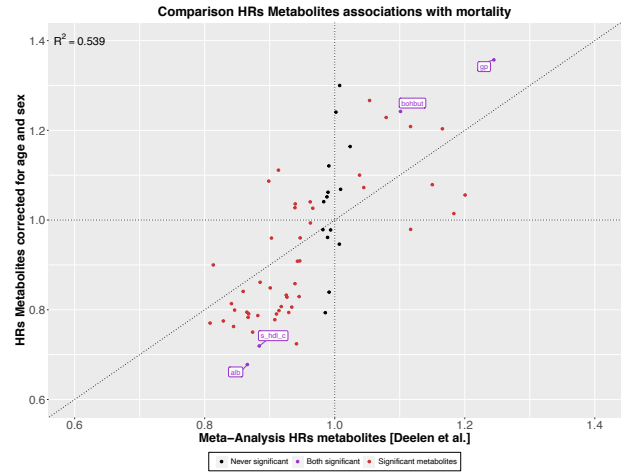

c

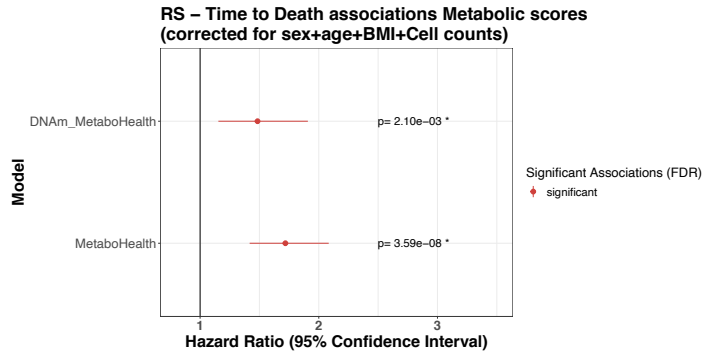

Supplement: Figure S8 — Mortality associations of the measured metabolomics in the 664 samples (99 deceased) of the Rotterdam Study with metabolomics and mortality data. a) Univariates associations with mortality of the metabolomics features divided in metabolomics groups. b) Comparison of the HRs of the metabolomics features in the RS with what previously reported by Deelen et al. c) Comparison of the univariate mortality associations with MetaboHealth and DNAm_MetaboHealth. On the right side of each forest plot there are the p values for each univariate association [cox regression]. [file mmc8.pdf]

a

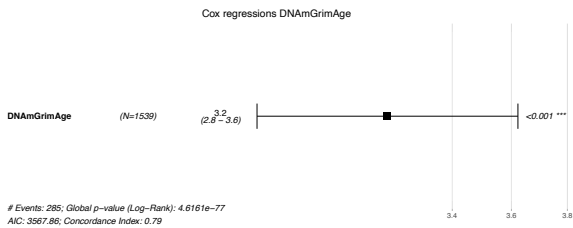

b

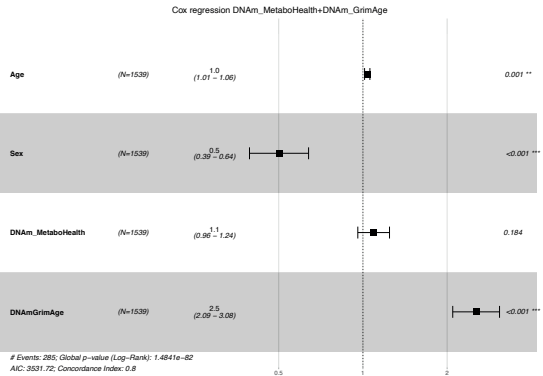

c

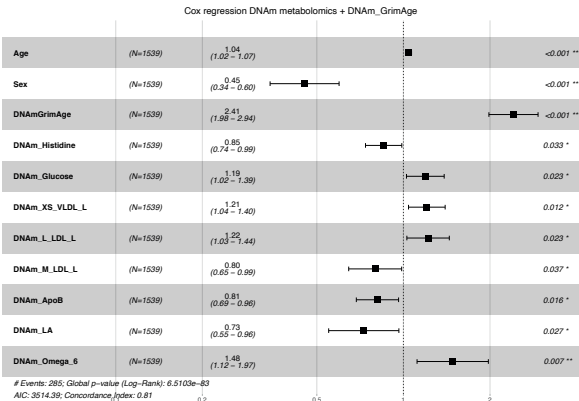

d

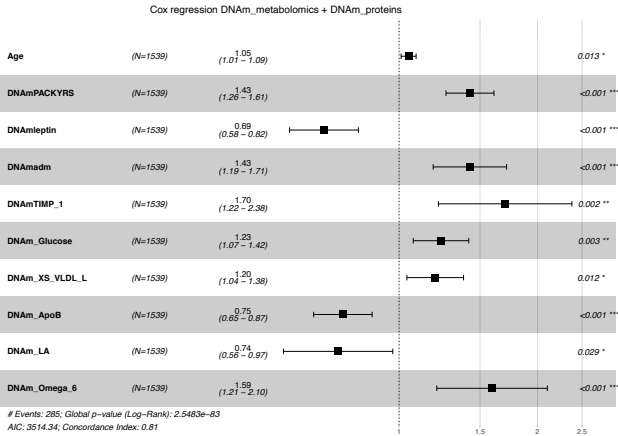

e

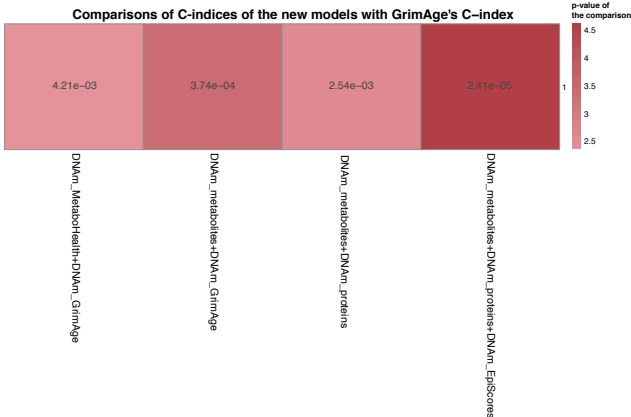

f

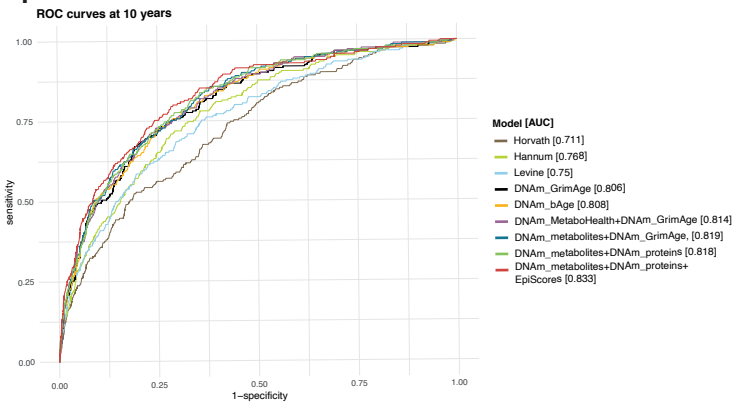

Supplement: Figure S10 — Multivariate mortality models built in the Rotterdam Study. a) Cox regression using GrimAge. b) Cox regression including age, sex, DNAm MetaboHealth score and GrimAge (DNAm_MetaboHealth+DNAm_GrimAge). c) Stepwise cox regression built with the DNAm metabolomics features and the GrimAge score (DNAm_metabolites+ DNAm_GrimAge). d) Stepwise cox regression built with the DNAm metabolomics features and the DNAm proteins surrogates included in GrimAge (DNAm_metabolites+ DNAm_proteins). e) p values evaluating the significance of the improvement in the C-indices of the newly developed multivariate mortality models compared with GrimAge. f) ROC curves and the accuracies (AUC) at 10-years mortality for our newly developed clocks as compared to previously trained scores. [file mmc10.pdf]

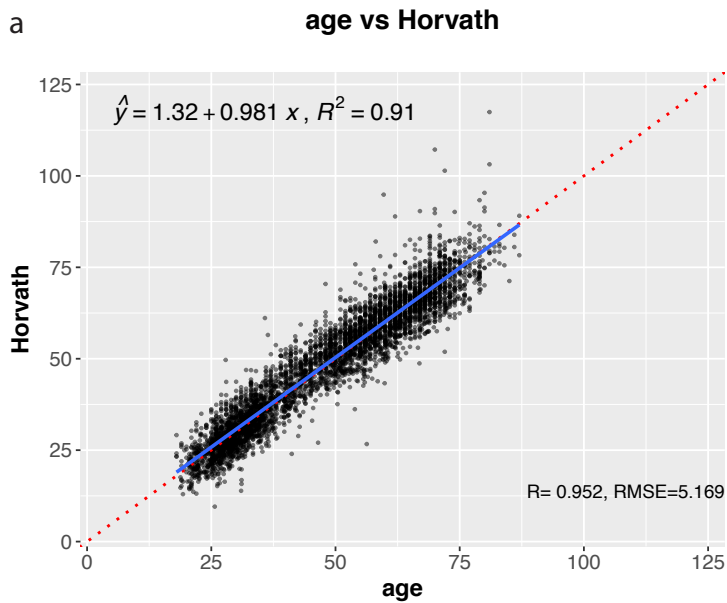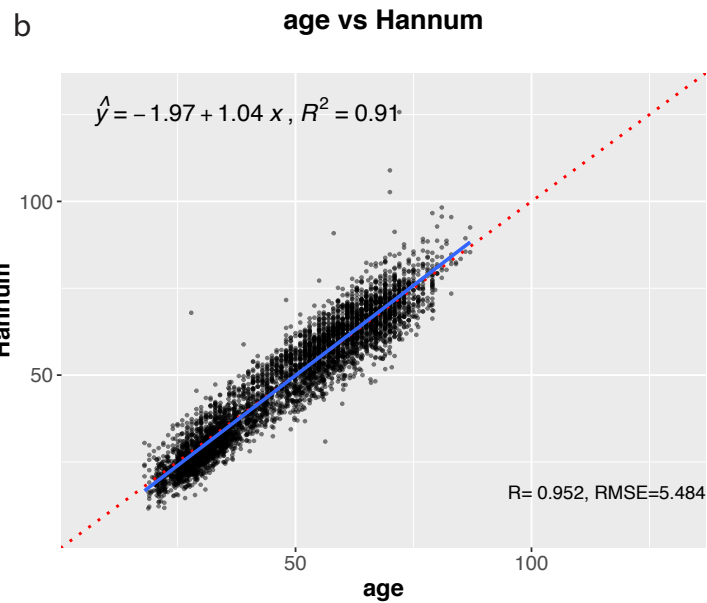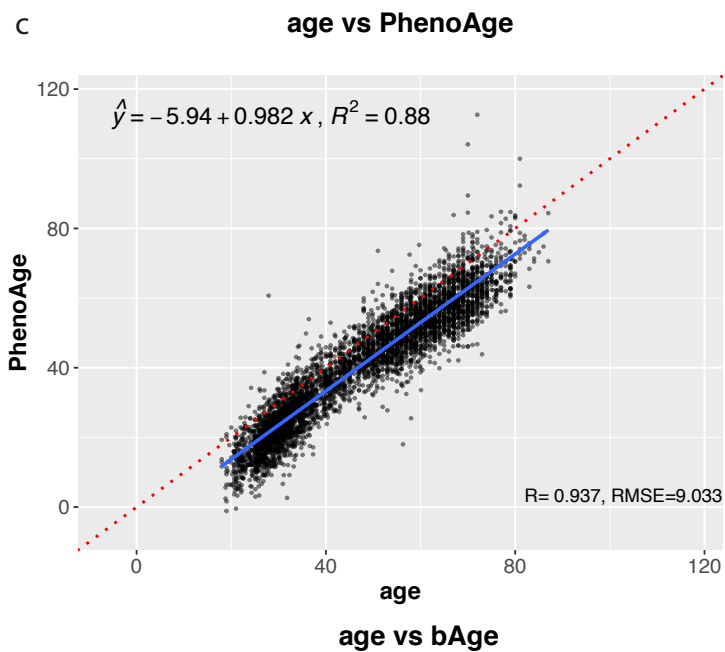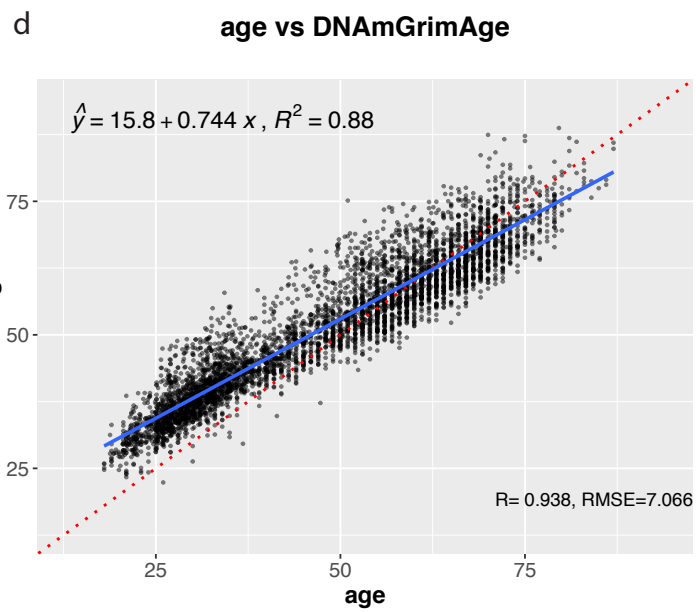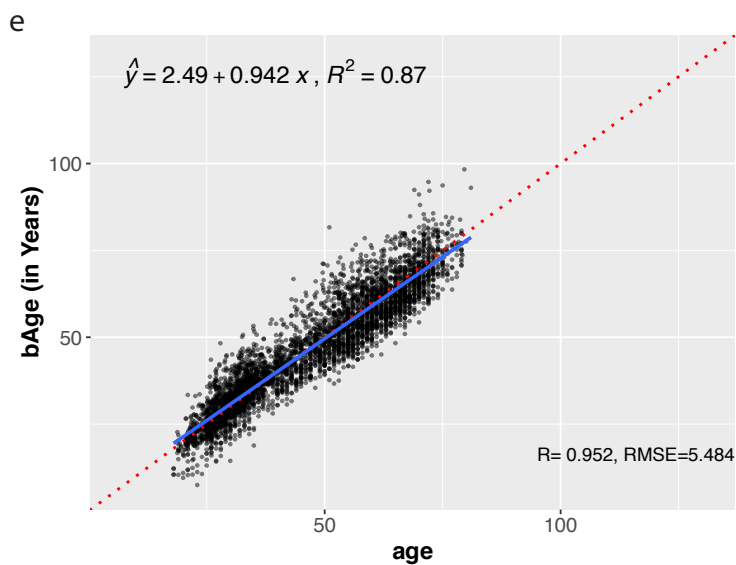

Supplement: Figure S13 — Correlation of the projected epigenetic clocks in BIOS. a) Horvath clock, b) Hannum, c) PhenoAge, d) GrimAge, and e) bAge. [file mmc13.pdf]

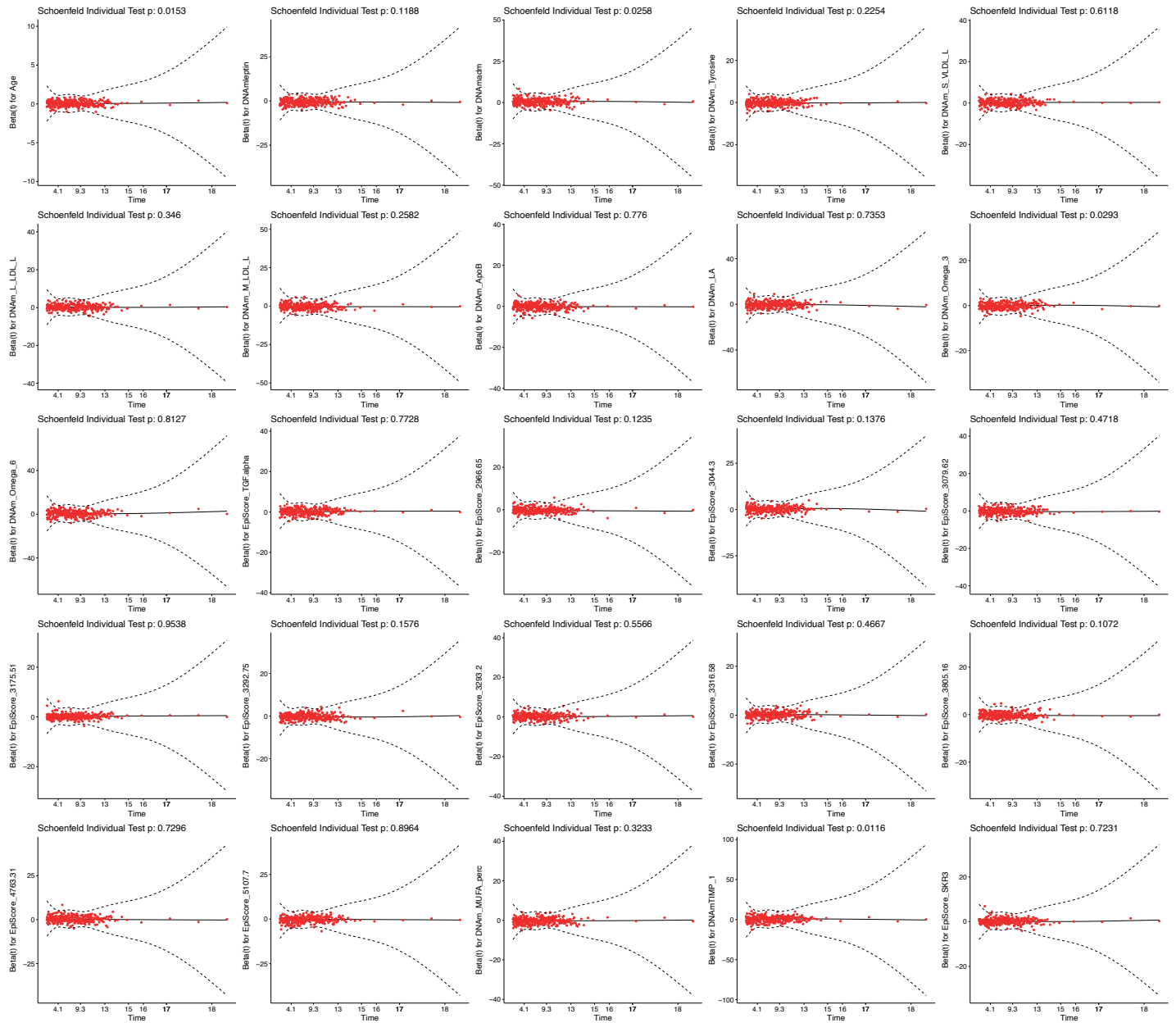

Supplement: Figure S14 — Schoenfield residuals for the model comprising DNAm metabolomics, GrimAge surrogates, and protein Episcores. Each figure represents the Schoenfield residuals for each of the features selected within our model, with the p value [Schoenfield] on the top, demonstrating the absence of patterns with time. [file mmc14.pdf]
