# Supplementary material for: NMR metabolomics-guided DNA methylation mortality predictors
Source: eBioMedicine. 2024 Aug 17;107:105279. doi: 10.1016/j.ebiom.2024.105279 (PMC11378104; doi:10.1016/j.ebiom.2024.105279)
Supplement: Figure S2 — Distributions of the matches used to perform the calibration on the metabolomics dataset. The number of matching samples between a) b) and c) are the age distributions of the matching samples. d), e) and f) are the BMI distributions of the matching samples. [file mmc2.pdf]

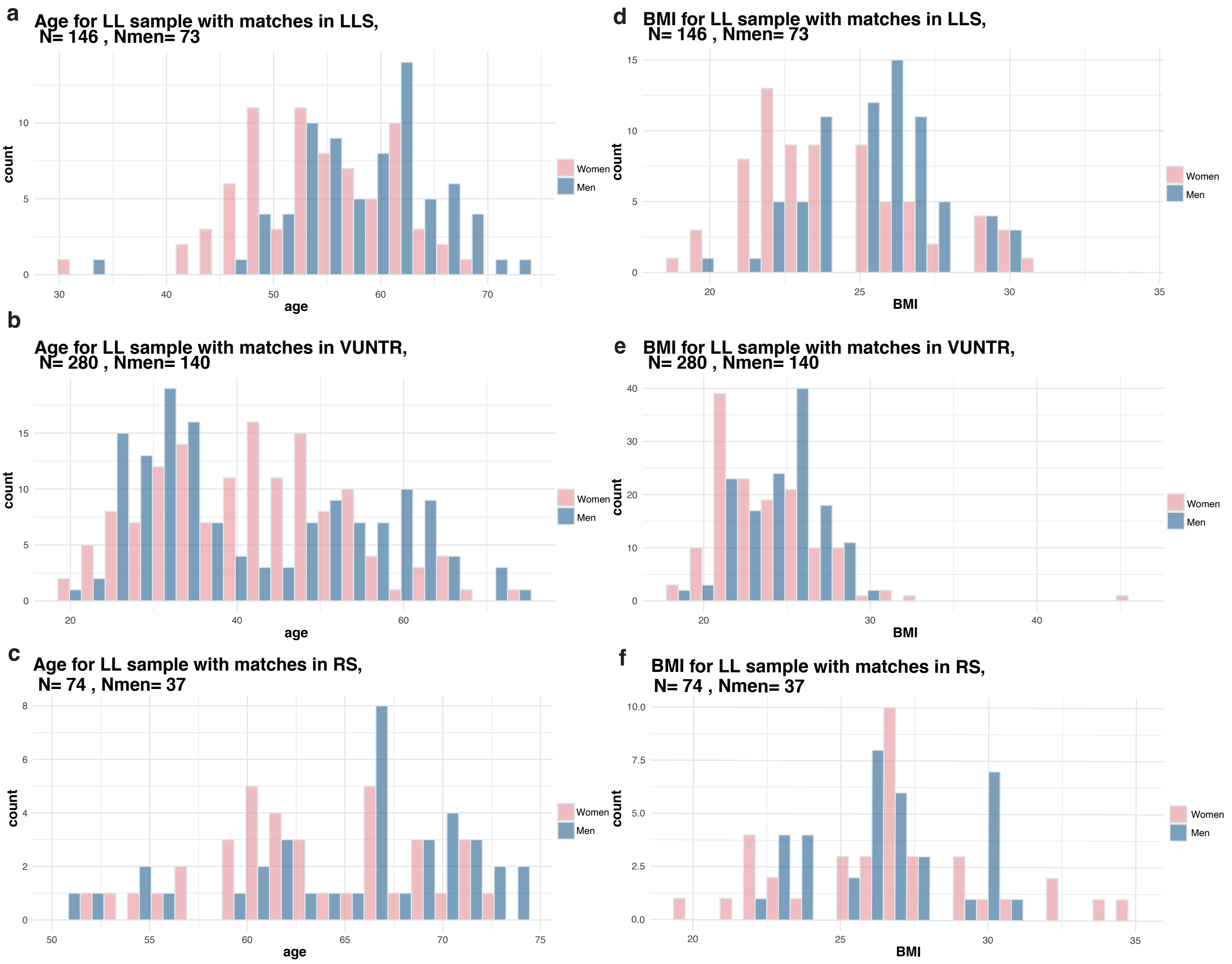
a
Age for LL sample with matches in LLS,
N= 146 , Nmen= 73
count
age
Women
Men
b
Age for LL sample with matches in VUNTR,
N= 280 , Nmen= 140
count
age
Women
Men
c
Age for LL sample with matches in RS,
N= 74 , Nmen= 37
count
age
Women
Men
d
BMI for LL sample with matches in LLS,
N= 146 , Nmen= 73
count
BMI
Women
Men
e
BMI for LL sample with matches in VUNTR,
N= 280 , Nmen= 140
count
BMI
Women
Men
f
BMI for LL sample with matches in RS,
N= 74 , Nmen= 37
count
BMI
Women
Men
