# Supplementary material for: NMR metabolomics-guided DNA methylation mortality predictors
Source: eBioMedicine. 2024 Aug 17;107:105279. doi: 10.1016/j.ebiom.2024.105279 (PMC11378104; doi:10.1016/j.ebiom.2024.105279)
Supplement: Figure S5 — DNAm metabolomics features accuracies divided in tertiles. Correlation of the DNAm metabolomics features with their quantified counterpart over the test sets (5Fold Cross-Validation test sets, LLS, and RS) divided in tertiles. Explicitly a) shows features with mean R >0.35, b) 0.2>mean R<0.35, c) mean R<0.2. [file mmc5.pdf]

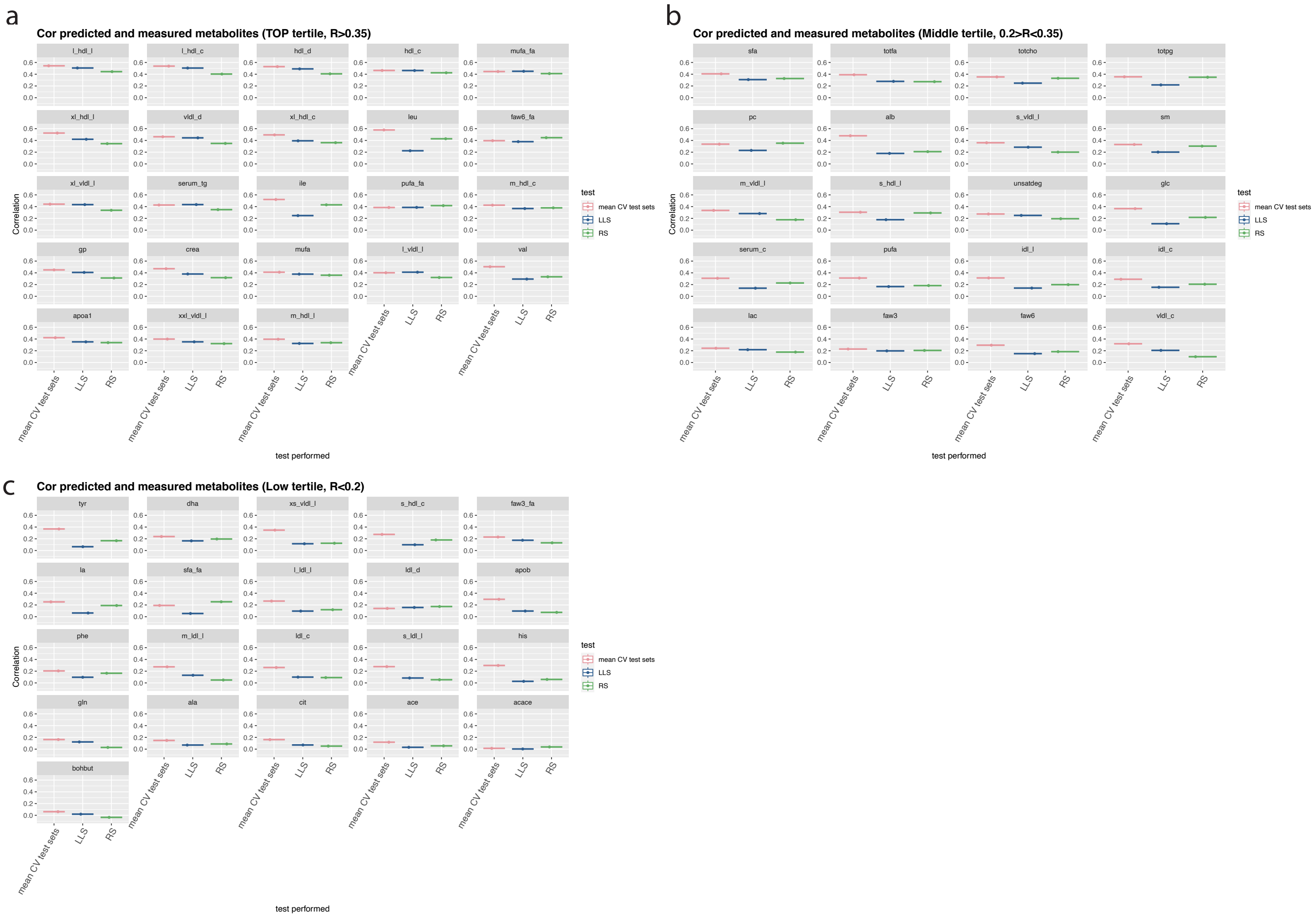
a
Cor predicted and measured metabolites (TOP tertile, R>0.35)
l_hdl_l
l_hdl_c
hdl_d
hdl_c
mufa_fa
xl_hdl_l
vldl_d
xl_hdl_c
leu
faw6_fa
xl_vldl_l
serum_tg
ile
pufa_fa
m_hdl_c
gp
crea
mufa
l_vldl_l
val
apoa1
xxl_vldl_l
m_hdl_l
Correlation
mean CV test sets
LLS
RS
test performed
test
b
Cor predicted and measured metabolites (Middle tertile, 0.2>R<0.35)
sfa
totfa
totcho
totpg
pc
alb
s_vldl_l
sm
m_vldl_l
s_hdl_l
unsatdeg
glc
serum_c
pufa
idl_l
idl_c
lac
faw3
faw6
vldl_c
c
Cor predicted and measured metabolites (Low tertile, R<0.2)
tyr
dha
xs_vldl_l
s_hdl_c
faw3_fa
la
sfa_fa
l_ldl_l
ldl_d
apob
phe
m_ldl_l
ldl_c
s_ldl_l
his
gln
ala
cit
ace
acace
bohbut
