# Supplementary material for: NMR metabolomics-guided DNA methylation mortality predictors
Source: eBioMedicine. 2024 Aug 17;107:105279. doi: 10.1016/j.ebiom.2024.105279 (PMC11378104; doi:10.1016/j.ebiom.2024.105279)
Supplement: Figure S9 — Univariate mortality associations in RS of the pre-trained scores. a) Univariate associations of each of the evaluated DNAm-based clocks (GrimAge, PhenoAge, Hannum and Horvath). b) The mortality associations with the DNAm surrogates included in GrimAge. c) The mortality association for each of the 109 plasma protein EpiScores. On the right side of each plot there are the p values for each univariate association [cox regression]. [file mmc9.pdf]

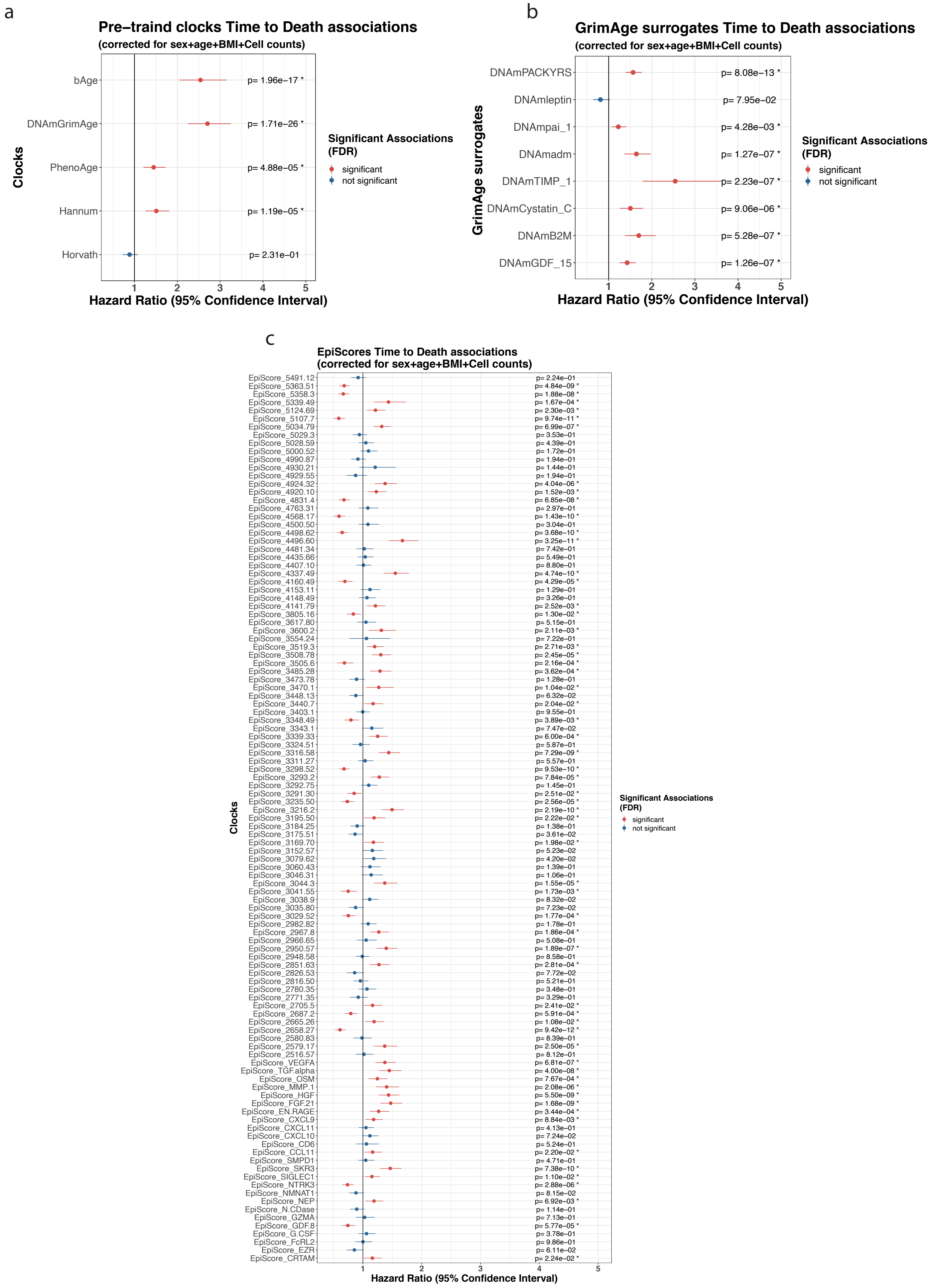
a
Pre–traind clocks Time to Death associations
(corrected for sex+age+BMI+Cell counts)
Clocks
bAge p= 1.96e–17 *
DNAmGrimAge p= 1.71e–26 *
PhenoAge p= 4.88e–05 *
Hannum p= 1.19e–05 *
Horvath p= 2.31e–01
1 2 3 4 5
Hazard Ratio (95% Confidence Interval)
Significant Associations (FDR)
significant
not significant
b
GrimAge surrogates Time to Death associations
(corrected for sex+age+BMI+Cell counts)
GrimAge surrogates
DNAmPACKYRS p= 8.08e–13 *
DNAmleptin p= 7.95e–02
DNAmpai_1 p= 4.28e–03 *
DNAmadm p= 1.27e–07 *
DNAmTIMP_1 p= 2.23e–07 *
DNAmCystatin_C p= 9.06e–06 *
DNAmB2M p= 5.28e–07 *
DNAmGDF_15 p= 1.26e–07 *
1 2 3 4 5
Hazard Ratio (95% Confidence Interval)
Significant Associations (FDR)
significant
not significant
c
EpiScores Time to Death associations
(corrected for sex+age+BMI+Cell counts)
Clocks
EpiScore_5491.12 p= 2.24e–01
EpiScore_5363.51 p= 4.84e–09 *
EpiScore_5358.3 p= 1.88e–08 *
EpiScore_5339.49 p= 1.67e–04 *
EpiScore_5124.69 p= 2.30e–03 *
EpiScore_5107.7 p= 9.74e–11 *
EpiScore_5034.79 p= 6.99e–07 *
EpiScore_5029.3 p= 3.53e–01
EpiScore_5028.59 p= 4.39e–01
EpiScore_5000.52 p= 1.72e–01
EpiScore_4990.87 p= 1.94e–01
EpiScore_4930.21 p= 1.44e–01
EpiScore_4929.55 p= 1.94e–01
EpiScore_4924.32 p= 4.04e–06 *
EpiScore_4920.10 p= 1.52e–03 *
EpiScore_4831.4 p= 6.85e–08 *
EpiScore_4763.31 p= 2.97e–01
EpiScore_4568.17 p= 1.43e–10 *
EpiScore_4500.50 p= 3.04e–01
EpiScore_4498.62 p= 3.68e–10 *
EpiScore_4496.60 p= 3.25e–11 *
EpiScore_4481.34 p= 7.42e–01
EpiScore_4435.66 p= 5.49e–01
EpiScore_4407.10 p= 8.80e–01
EpiScore_4337.49 p= 4.74e–10 *
EpiScore_4160.49 p= 4.29e–05 *
EpiScore_4153.11 p= 1.29e–01
EpiScore_4148.49 p= 3.26e–01
EpiScore_4141.79 p= 2.52e–03 *
EpiScore_3805.16 p= 1.30e–02 *
EpiScore_3617.80 p= 5.15e–01
EpiScore_3600.2 p= 2.11e–03 *
EpiScore_3554.24 p= 7.22e–01
EpiScore_3519.3 p= 2.71e–03 *
EpiScore_3508.78 p= 2.45e–05 *
EpiScore_3505.6 p= 2.16e–04 *
EpiScore_3485.28 p= 3.62e–04 *
EpiScore_3473.78 p= 1.28e–01
EpiScore_3470.1 p= 1.04e–02 *
EpiScore_3448.13 p= 6.32e–02
EpiScore_3440.7 p= 2.04e–02 *
EpiScore_3403.1 p= 9.55e–01
EpiScore_3348.49 p= 3.89e–03 *
EpiScore_3343.1 p= 7.47e–02
EpiScore_3339.33 p= 6.00e–04 *
EpiScore_3324.51 p= 5.87e–01
EpiScore_3316.58 p= 7.29e–09 *
EpiScore_3311.27 p= 5.57e–01
EpiScore_3298.52 p= 9.53e–10 *
EpiScore_3293.2 p= 7.84e–05 *
EpiScore_3292.75 p= 1.45e–01
EpiScore_3291.30 p= 2.51e–02 *
EpiScore_3235.50 p= 2.56e–05 *
EpiScore_3216.2 p= 2.19e–10 *
EpiScore_3195.50 p= 2.22e–02 *
EpiScore_3184.25 p= 1.38e–01
EpiScore_3175.51 p= 3.61e–02
EpiScore_3169.70 p= 1.98e–02 *
EpiScore_3152.57 p= 5.23e–02
EpiScore_3079.62 p= 4.20e–02
EpiScore_3060.43 p= 1.39e–01
EpiScore_3046.31 p= 1.06e–01
EpiScore_3044.3 p= 1.55e–05 *
EpiScore_3041.55 p= 1.73e–03 *
EpiScore_3038.9 p= 8.32e–02
EpiScore_3035.80 p= 7.23e–02
EpiScore_3029.52 p= 1.77e–04 *
EpiScore_2982.82 p= 1.78e–01
EpiScore_2967.8 p= 1.86e–04 *
EpiScore_2966.65 p= 5.08e–01
EpiScore_2950.57 p= 1.89e–07 *
EpiScore_2948.58 p= 8.58e–01
EpiScore_2851.63 p= 2.81e–04 *
EpiScore_2826.53 p= 7.72e–02
EpiScore_2816.50 p= 5.21e–01
EpiScore_2780.35 p= 3.48e–01
EpiScore_2771.35 p= 3.29e–01
EpiScore_2705.5 p= 2.41e–02 *
EpiScore_2687.2 p= 5.91e–04 *
EpiScore_2665.26 p= 1.08e–02 *
EpiScore_2658.27 p= 9.42e–12 *
EpiScore_2580.83 p= 8.39e–01
EpiScore_2579.17 p= 2.50e–05 *
EpiScore_2516.57 p= 8.12e–01
EpiScore_VEGFA p= 6.81e–07 *
EpiScore_TGF.alpha p= 4.00e–08 *
EpiScore_OSM p= 7.67e–04 *
EpiScore_MMP.1 p= 2.08e–06 *
EpiScore_HGF p= 5.50e–09 *
EpiScore_FGF.21 p= 1.68e–09 *
EpiScore_EN.RAGE p= 3.44e–04 *
EpiScore_CXCL9 p= 8.84e–03 *
EpiScore_CXCL11 p= 4.13e–01
EpiScore_CXCL10 p= 7.24e–02
EpiScore_CD6 p= 5.24e–01
EpiScore_CCL11 p= 2.20e–02 *
EpiScore_SMPD1 p= 4.71e–01
EpiScore_SKR3 p= 7.38e–10 *
EpiScore_SIGLEC1 p= 1.10e–02 *
EpiScore_NTRK3 p= 2.88e–06 *
EpiScore_NMNAT1 p= 8.15e–02
EpiScore_NEP p= 6.92e–03 *
EpiScore_N.CDase p= 1.14e–01
EpiScore_GZMA p= 7.13e–01
EpiScore_GDF.8 p= 5.77e–05 *
EpiScore_G.CSF p= 3.78e–01
EpiScore_FcRL2 p= 9.86e–01
EpiScore_EZR p= 6.11e–02
EpiScore_CRTAM p= 2.24e–02 *
1 2 3 4 5
Hazard Ratio (95% Confidence Interval)
Significant Associations (FDR)
significant
not significant
