# Supplementary material for: NMR metabolomics-guided DNA methylation mortality predictors
Source: eBioMedicine. 2024 Aug 17;107:105279. doi: 10.1016/j.ebiom.2024.105279 (PMC11378104; doi:10.1016/j.ebiom.2024.105279)

Percentage of overlap the cpgs used in each DNAm model

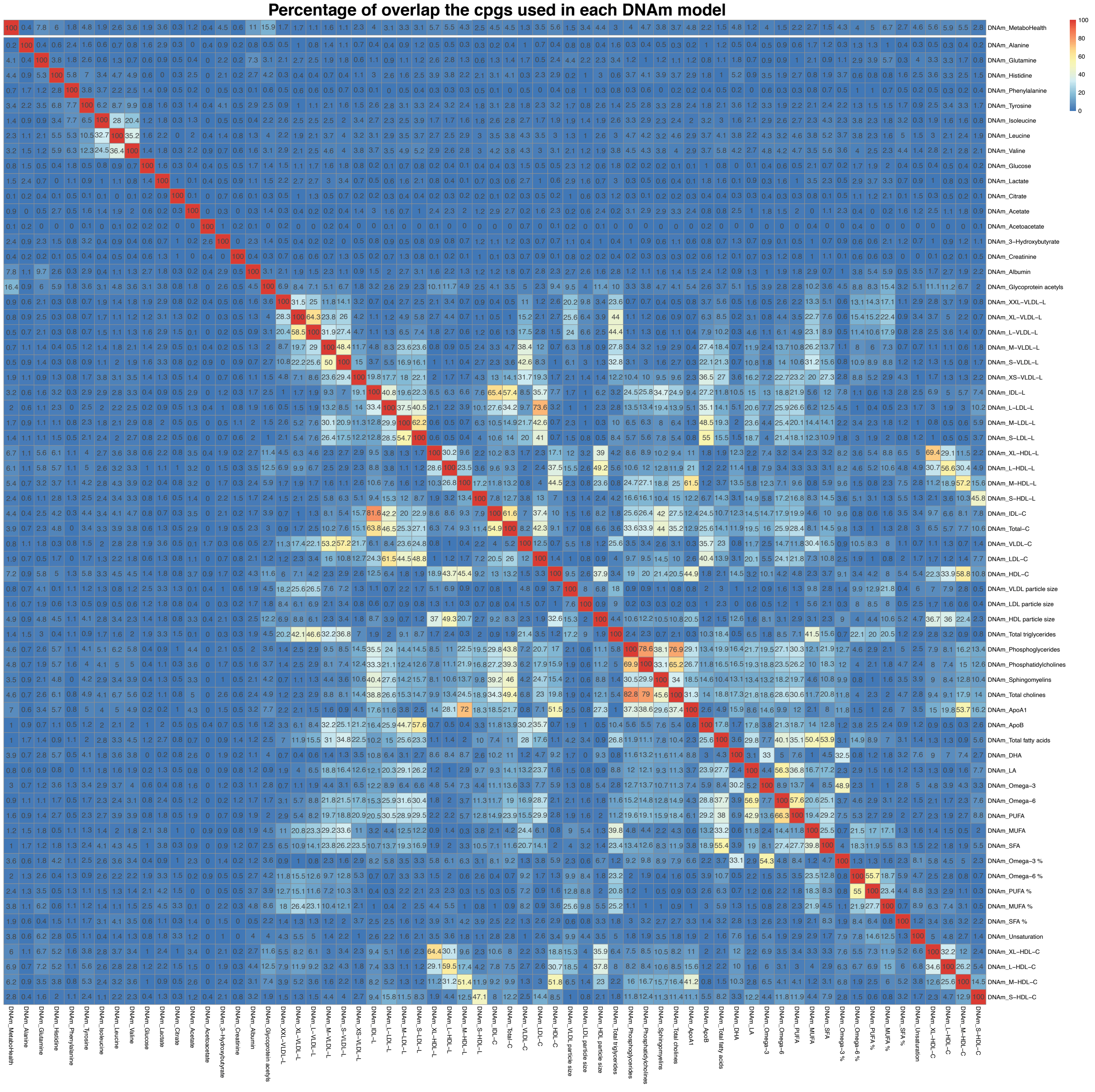

Supplement: Figure S12 — Percentage overlap of the CpG sites selected by each DNAm model. [file mmc12.pdf]
