# Supplementary material for: NMR metabolomics-guided DNA methylation mortality predictors
Source: eBioMedicine. 2024 Aug 17;107:105279. doi: 10.1016/j.ebiom.2024.105279 (PMC11378104; doi:10.1016/j.ebiom.2024.105279)
Supplement: BBMRI-consortium-author [file mmc16.docx]

## BBMRI Consortium author list

| **Initials** | **Surname** |
| --- | --- |
| J.M. | Geleijnse |
| E. | Boersma |
| W.E. | van Spil |
| M.M.J. | van Greevenbroek |
| C.D.A. | Stehouwer |
| C.J.H. | van der Kallen |
| I.C.W. | Arts |
| F. | Rutters |
| J.W.J. | Beulens |
| M. | Muilwijk |
| P.J.M. | Elders |
| L.M. | 't Hart |
| M. | Ghanbari |
| M.A. | Ikram |
| M.G. | Netea |
| M. | Kloppenburg |
| Y.F.M. | Ramos |
| N. | Bomer |
| I. | Meulenbelt |
| K. | Stronks |
| M.B. | Snijder |
| A.H. | Zwinderman |
| B.T. | Heijmans |
| L.H. | Lumey |
| C. | Wijmenga |
| J. | Fu |
| A. | Zhernakova |
| J. | Deelen |
| S.P. | Mooijaart |
| M. | Beekman |
| P.E. | Slagboom |
| G.L.J. | Onderwater |
| A.M.J.M. | van den Maagdenberg |
| G.M. | Terwindt |
| C. | Thesing |
| M. | Bot |
| B.W.J.H. | Penninx |
| S. | Trompet |
| J.W. | Jukema |
| N. | Sattar |
| I.C.C. | van der Horst |
| P. | van der Harst |
| C. | So-Osman |
| J.A. | van Hilten |
| R.G.H.H. | Nelissen |
| I.E. | Höfer |
| F.W. | Asselbergs |
| P. | Scheltens |
| C.E. | Teunissen |
| W.M. | van der Flier |
| J. | van Dongen |
| R. | Pool |
| A.H.M. | Willemsen |
| D.I. | Boomsma |

## BBMRI Consortium Banner


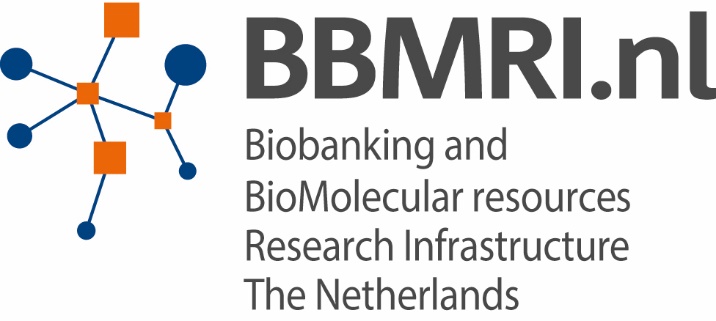
METABOLOMICS CONSORTIUM

Cohort Collection:

J.M. Geleijnse^1^, E. Boersma^2^, W.E. van Spil^3^, M.M.J. van Greevenbroek^4, 5^, C.D.A. Stehouwer^4, 5^, C.J.H. van der Kallen^4, 5^, I.C.W. Arts^5, 6, 7^, F. Rutters^8, 9^, J.W.J. Beulens^8, 9^, M. Muilwijk^8, 10^, P.J.M. Elders^8, 10^, L.M. 't Hart^8, 9, 11, 12^, M. Ghanbari^13, 14^, M.A. Ikram^13^, M.G. Netea^15^, M. Kloppenburg^16, 17^, Y.F.M. Ramos^18^, N. Bomer^19^, I. Meulenbelt^18^, K. Stronks^20^, M.B. Snijder^20^, A.H. Zwinderman^21^, B.T. Heijmans^18^, L.H.Lumey^22^, C. Wijmenga^23^, J. Fu^23, 24^, A. Zhernakova^23^, J. Deelen^25, 18^, S.P. Mooijaart^26^, M. Beekman^18^, P.E. Slagboom^18, 25^, G.L.J. Onderwater^27^, A.M.J.M. van den Maagdenberg^28, 27^, G.M. Terwindt^27^, C.Thesing^29, 8^, M. Bot^29, 8^, B.W.J.H. Penninx^29, 8^, S. Trompet^30, 26^, J.W. Jukema^30^, N. Sattar^31^, I.C.C. van der Horst^32^, P. van der Harst^33^, C. So-Osman^34, 35^, J.A. van Hilten^36^, R.G.H.H. Nelissen^37^, I.E. Höfer^38^, F.W. Asselbergs^39, 40^, P. Scheltens^41^, C.E. Teunissen^42^, W.M. van der Flier^43, 41^, J. van Dongen^29, 8^, R. Pool^29^, A.H.M. Willemsen^29, 8^, D.I. Boomsma^29, 8^

Sample Logistics, Database & Catalogue:

H.E.D. Suchiman^18^, J.J.H. Barkey Wolf^18^, M. Beekman^18^, D. Cats^45^, H. Mei^45^, M. Slofstra^23^, M. Swertz^46, 23^, M.J.T. Reinders^47, 48^, E.B. van den Akker^47, 18^

Steering committee:

D.I. Boomsma^29, 8^, M.A. Ikram^13^, P.E. Slagboom^18, 25^

Affiliations:

1. Division of Human Nutrition and Health, Wageningen University, Wageningen, The Netherlands
2. Thorax centre, Erasmus Medical Centre, Rotterdam, the Netherlands
3. Department of Rheumatology & Clinical Immunology, University Medical Center Utrecht, Utrecht, The Netherlands
4. Department of Internal Medicine, Maastricht University Medical Center (MUMC+), Maastricht, The Netherlands
5. School for Cardiovascular Diseases (CARIM), Maastricht University, Maastricht, the Netherlands
6. Department of Epidemiology, Maastricht University, Maastricht, the Netherlands
7. Maastricht Center for Systems Biology, Maastricht University, Maastricht, the Netherlands
8. Amsterdam Public Health Research Institute, Amsterdam, The Netherlands
9. Department of Epidemiology and Biostatistics, Amsterdam University Medical Center, Vrije Universiteit, Amsterdam, the Netherlands
10. Department of General Practice and Elderly Care Medicine, Amsterdam University Medical Center, Vrije Universiteit, Amsterdam, the Netherlands
11. Department of Epidemiology and Biostatistics, Amsterdam University Medical Center, Vrije Universiteit, Amsterdam, the Netherlands
12. Department of Cell and Chemical Biology, Leiden University Medical Center, Leiden, the Netherlands
13. Department of Epidemiology, Erasmus MC, University Medical Center, Rotterdam, The Netherlands
14. Department of Genetics, School of Medicine,, Mashhad University of Medical Sciences, Mashhad, Iran
15. Department of Internal Medicine and Radboud Center for Infectious Diseases, Radboud University Medical Center, Nijmegen, The Netherlands
16. Department of Clinical Epidemiology, Leiden University Medical Centre, Leiden, The Netherlands
17. Department of Rheumatology, Leiden University Medical Center, The Netherlands
18. Department of Biomedical Data Sciences, Section of Molecular Epidemiology, Leiden University Medical Center, Leiden, The Netherlands
19. Department of Experimental Cardiology, University of Groningen, University Medical Center Groningen, Groningen, The Netherlands
20. Department of Public Health, Academic Medical Center, University of Amsterdam, Amsterdam, The Netherlands
21. Department of Clinical Epidemiology, Biostatistics, and Bioinformatics, Academic Medical Centre, University of Amsterdam, Amsterdam, The Netherlands
22. Department of Epidemiology, Mailman School of Public Health, Columbia University, New York, NY 10032
23. Department of Genetics, University Medical Center Groningen, Groningen, The Netherlands
24. Department of Pediatrics, University Medical Center Groningen, Groningen, The Netherlands
25. Max Planck Institute for Biology of Ageing, Cologne, Germany
26. Department of Internal Medicine, Division of Gerontology and Geriatrics, Leiden University Medical Centre, Leiden, The Netherlands
27. Department of Neurology, Leiden University Medical Center, Leiden, The Netherlands
28. Department of Human Genetics, Leiden University Medical Center, Leiden, The Netherlands
29. Department of Biological Psychology, Amsterdam University Medical Center, Vrije Universiteit, Amsterdam, The Netherlands
30. Department of Cardiology, Leiden University Medical Center, Leiden, The Netherlands
31. Institute of Cardiovascular and Medical Sciences, Cardiovascular Research Centre, University of Glasgow, Glasgow, UK
32. Department of Critical Care, University Medical Center Groningen, Groningen, The Netherlands
33. Department of Cardiology, University Medical Center Utrecht, Utrecht, The Netherlands
34. Sanquin Blood Bank, Leiden and Department of Haematology, Groene Hart Hospital, Gouda, the Netherlands
35. International Society of Blood Transfusion (ISBT), Amsterdam, The Netherlands
36. Unit of Transfusion Medicine, Sanquin Blood Bank, Leiden, The Netherlands
37. Department of Orthopaedics, Leiden University Medical Center, Leiden, The Netherlands
38. Department of Clinical Chemistry and Hematology, UMC Utrecht, the Netherlands
39. Department of Cardiology, Division Heart and Lungs, University Medical Center Utrecht, Utrecht, The Netherlands
40. Julius Center for Health Sciences and Primary Care, University Medical Center Utrecht, Utrecht, The Netherlands
41. Department of Neurology & Alzheimer Center, VU University Medical Center, Amsterdam, The Netherlands
42. Neurochemistry Laboratory, Clinical Chemistry Department, Amsterdam University Medical Center, Amsterdam Neuroscience, The Netherlands
43. Department of Epidemiology and Biostatistics, VU University Medical Center, Amsterdam, The Netherlands
44. SURFsara, Amsterdam, the Netherlands
45. Sequence Analysis Support Core, Leiden University Medical Center, Leiden, the Netherlands
46. University of Groningen, University Medical Center Groningen, Genomics Coordination Center, Groningen, the Netherlands
47. Leiden Computational Biology Center, Leiden University Medical Center, Leiden, the Netherlands

The Delft Bioinformatics Lab, Delft University of Technology, Delft, the Netherlands
