# Supplementary material for: NMR metabolomics-guided DNA methylation mortality predictors
Source: eBioMedicine. 2024 Aug 17;107:105279. doi: 10.1016/j.ebiom.2024.105279 (PMC11378104; doi:10.1016/j.ebiom.2024.105279)
Supplement: Figure S11 — Enrichment analysis of the CpGs selected by DNAm models. a) Log10 p value [fisher test] of all the significant associations in the enrichment analysis over the selected CpG in the EWAS Catalog and Atlas. b) Median coefficients of the CpGs in the DNAm metabolomics models for the overlapping CpGs with the first 50 significantly enriched traits in the EWAS Catalog and Atlas. c) Log10 p value [fisher test] of the first 50 Gene Ontology traits enriched with the nearest genes to the selected CpG sites. [file mmc11.pdf]

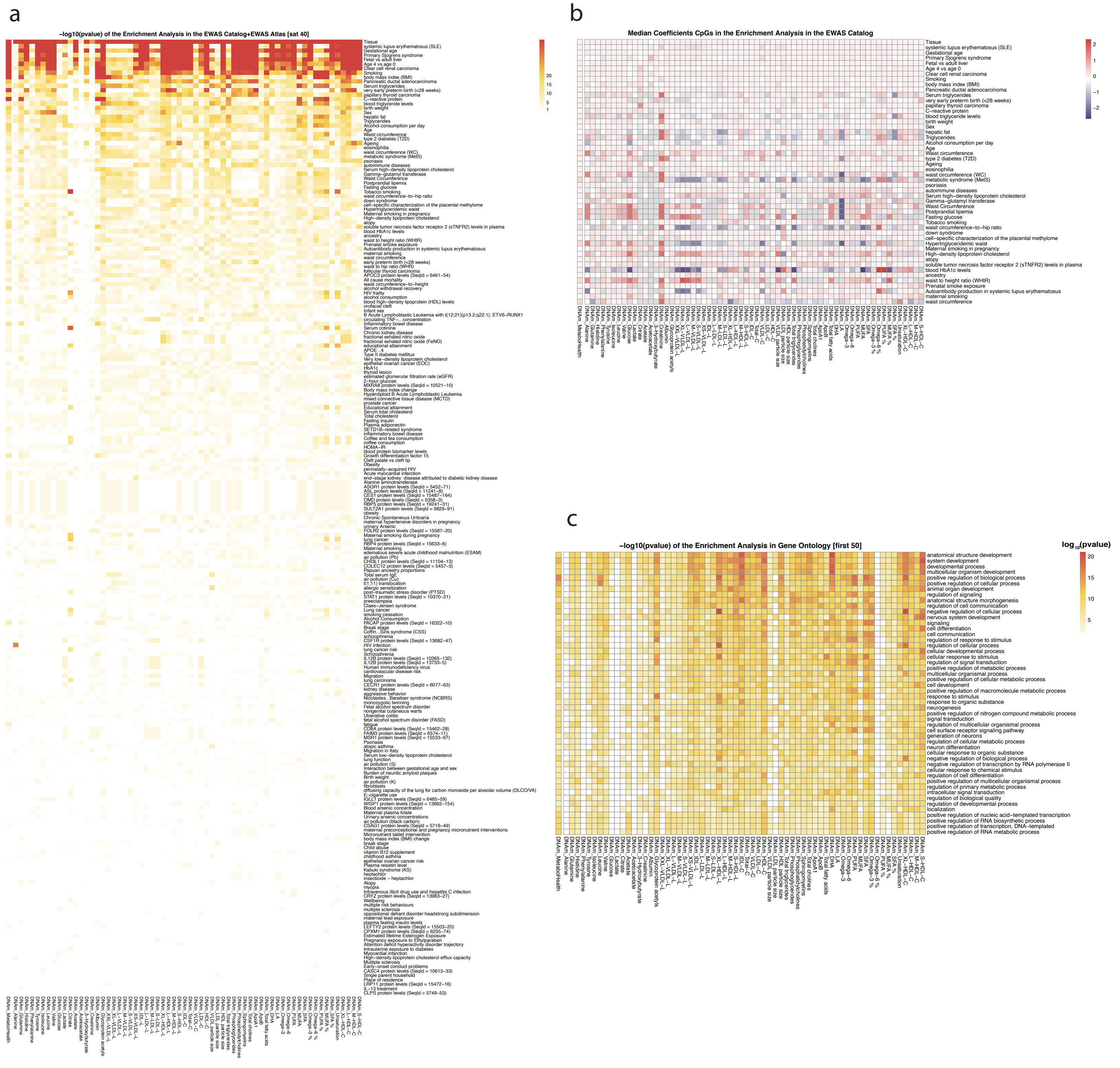
a
−log10(pvalue) of the Enrichment Analysis in the EWAS Catalog+EWAS Atlas [sat 40]
b
Median Coefficients CpGs in the Enrichment Analysis in the EWAS Catalog
c
−log10(pvalue) of the Enrichment Analysis in Gene Ontology [first 50]
log10(pvalue)
